# Hybrid modeling as a QbD/PAT tool in process development: an industrial *E. coli* case study

**DOI:** 10.1007/s00449-016-1557-1

**Published:** 2016-02-15

**Authors:** Moritz von Stosch, Jan-Martijn Hamelink, Rui Oliveira

**Affiliations:** CEAM, Faculty of Science, Agriculture and Engineering, Newcastle University, Newcastle upon Tyne, NE1 7RU UK; REQUIMTE/DQ, Faculty of Science and Technology, University Nova de Lisboa, Campus de Caparica, 2829-516 Caparica, Portugal; GSK Vaccines, Laval, Canada; Lallemand Inc., Montreal, Canada

**Keywords:** Upstream bioprocess development/optimization, Dynamic modeling, Hybrid modeling, *E. coli*, High cell density fermentation

## Abstract

Process understanding is emphasized in the process analytical technology initiative and the quality by design paradigm to be essential for manufacturing of biopharmaceutical products with consistent high quality. A typical approach to developing a process understanding is applying a combination of design of experiments with statistical data analysis. Hybrid semi-parametric modeling is investigated as an alternative method to pure statistical data analysis. The hybrid model framework provides flexibility to select model complexity based on available data and knowledge. Here, a parametric dynamic bioreactor model is integrated with a nonparametric artificial neural network that describes biomass and product formation rates as function of varied fed-batch fermentation conditions for high cell density heterologous protein production with *E. coli*. Our model can accurately describe biomass growth and product formation across variations in induction temperature, pH and feed rates. The model indicates that while product expression rate is a function of early induction phase conditions, it is negatively impacted as productivity increases. This could correspond with physiological changes due to cytoplasmic product accumulation. Due to the dynamic nature of the model, rational process timing decisions can be made and the impact of temporal variations in process parameters on product formation and process performance can be assessed, which is central for process understanding.

## Introduction

Bioprocess development and optimization are essential elements of the biopharmaceutical business model and manufacturing economics. Robust process design is desired early on, since process changes at a later stage often require re-approval by regulatory authorities [1]. At the level of product manufacturing, United States’ Food and Drug Administration (FDA) has introduced the Process Analytical Technology (PAT) initiative intended to change from “recipe” production and off-line testing to real-time on-line testing and closed-loop control of intermediates and end product(s) [2]. A related strategic approach to quality product development is quality by design (QbD) [3–5]. The basis of QbD is to understand the sources of variability in process and product and to understand the linkages so that variability can be controlled [3–7].

Bioprocesses are often affected by a large set of input and output parameters of which the critical process parameters (CPPs) and critical quality attributes (CQAs) are the parameters to identify and assess [7, 8]. An integral QbD/PAT tool to determine the effects of multivariate interactions is statistical design of experiments (DoE) [9–12]. Application of a DoE strategy provides understanding of the relationship between parameters and CQAs and leads to establishment of a design space and ultimately a control space [3–5, 9, 13]. The control space defines the operational limits of the CPPs such that the quality of the CQAs can be ensured [10, 13].

Data obtained from the execution of DoEs are usually analyzed using statistical data analysis and regression models [9, 14–16]. In most situations, response surface models (RSM) are used to determine interactions of parameters in the design space [15, 17–19]. However, these approaches disregard mechanistic knowledge of the process. More classical modeling approaches seek to describe the process using first-principles, but have difficulty to accurately describe complex processes without laborious development [3, 4, 20, 21]. An alternative to these approaches is hybrid semi-parametric modeling [4, 20–23], hereupon shortly referred to as hybrid modeling. Hybrid modeling allows incorporating existing process knowledge and experimental data into a flexible framework in which both parametric (model structure specified by fundamental knowledge) and non-parametric model (model structure identified from data) structures are working together. Additional knowledge and data can be added to the model framework as they become available. Several authors have described hybrid models of upstream bioprocesses, in which knowledge and data related to the biological system were added to a bioreactor model [24, 25]. Hybrid models have been used for the modeling, monitoring or optimization of industrial fermentations [26–30]. Their benefits for PAT have been assessed [4, 21, 26, 27], although an industrial bioprocess development case, in particular with respect to PAT and QbD, has not yet been reported.

The purpose of this work is to study the application of hybrid modeling to a dataset generated during a DoE investigation to find optimal induction conditions for recombinant protein expression in *E. coli*. In the developed hybrid model, a general parametric bioreactor model was integrated with a nonparametric artificial neural network to correlate biomass and product formation rates with process parameters. Model and process performance are assessed and discussed with respect to PAT and QbD.

## Methods

### Fermentation process

The *E. coli* fermentation process development batches are conducted in four identical fermenters. Cultivations are started with a constant volume of batch medium. Fixed aeration rate and vessel pressures are applied, while dissolved oxygen is regulated by agitation speed. Batches are inoculated at identical biomass concentrations.

The fermentation process consists of three phases: (1) growth in batch mode, (2) growth in fed-batch mode; and (3) induction of product expression by addition of IPTG. Temperature is regulated through water jacket control and pH is controlled by base addition. Exhaustion of carbon source at the end of the batch phase is indicated by a pH rise and agitation drop [31, 32]. At this point, the fed-batch phase is initiated (automated step). Feed of fed-batch medium is regulated through gravimetric feed control. Up to this point, batch and fed-batch conditions are identical in all fermentations.

Cytoplasmic expression of the recombinant protein occurs after induction with IPTG. Four induction phase parameters were examined through DoE to identify optimal expression conditions: induction temperature, pH, feed rate and biomass concentration at induction. All other conditions were kept constant. The total induction period varied from 22 to 30 h in different batches, but all batches were sampled at least at the 22 h time point, which was selected as experimental end-point of the DoE.

### On-line measurements

On-line process parameters (temperature, pH, dissolved oxygen, agitation rate, air-flow rate, vessel pressure and feed and base balance readings) were recorded at 15 s intervals. Principally used for real-time feed-back control loops, the recorded on-line data is otherwise typically under-utilized, only being employed for batch profile generation, qualitative batch-to-batch comparisons and occasional basic data analysis.

For hybrid modeling the dataset treatment was as follows. The high frequency on-line data of each batch was averaged in 30 min intervals to increase hybrid modeling computing efficiency. The data starting point (*t*_0_) was selected at the first sample point during fed-batch phase (often the pre-induction sample). Each data file is then continued until end-of-batch in 30 min steps.

### Biomass, product and metabolite quantification

In-process samples were taken manually at induction and at the pre-defined end-point of 22 h of induction. Over the course of the batches, a small number of additional samples (up to a maximum of 6) were taken during fed-batch and induction phases.

Biomass concentration was determined by measurement of optical density (OD) at 650 nm (analytical error: 2–3 %). OD and dry cell weight (DCW) correlate well in a linear relationship: DCW (g/L) = 0.6 × OD (*R*^2^ = 0.98, *n* = 97 pre- and post-induction samples). For quantification of the soluble cytoplasmic expressed product, in-process samples are mechanically lysed using a bead mill homogenizer to produce three separate fractions: total fraction, pellet (insoluble fraction) and supernatant (soluble fraction). An assessment of productivity on all three fractions was performed by densitometry of Coomassie stained SDS-PAGE which revealed complete product solubility. Productivity results mentioned in this work were produced by an orthogonal quantification method that is based on RP-HPLC analysis of further treated supernatant samples against purified standards (analytical error: 5–10 %, based on repetitions starting from unfractionated culture samples). Volumetric productivity results of this RP-HPLC assay, normally expressed in mg/l, were scaled and made dimensionless for confidentiality reasons.

Sporadically, supernatants from culture samples were also analyzed for acetate content using a Bioprofile 300 analyzer from Nova Biomedical. However, the analytical error of these measurements is unknown but considered to be relatively high.

### Process development batches

After initial screening batches, a total of 53 fermentations batches were conducted to optimize productivity, either part of a DoE design, a set of investigative batches or part of a set of fermentations to confirm reproducibility. Table 1 summarizes the 53 batches, their respective design structures and the investigated conditions. Selection of each design structure, its factors and their analytical ranges, were result of a procedure in which previous experience with product and process, results of screening experiments and business related constraints were considered. Process temperature, pH and feed rate were scaled to preserve process confidentiality.

**Table 1:** Description of design of experiments, fermentation process conditions and hybrid modeling data sets

Dataset # and description		Design	Design descriptio (number of levels)	Factor ranges						Hybrid model dataset 1			Hybrid model dataset 2		
				*X*_Ind_ (OD)	*T* (−)	pH (−)	*u*_*C*_ (−)	#	Comments	tr	val	tst	tr	val	tst
1	DoE 1 batches	Doehlert	Four factors: *T*(7)/pH(5)/OD(5)/*F*(3)	38–78	−0.9 to 0.9	−0.9 to 0.9	−0.8 to 0.8	23	Includes three center point batches	15	8	–	15	4	4
2	Failed DoE 1 batches			58–70	−0.5 to 0.5	−0.1 to 0.9	−1.3 to 4.5	4	Constant *T* and pH; variable *u*_*C*_.	–	–	4	–	–	4
3	Investigative batch at optimal DoE 1 conditions			43	−0.8	0.0	0.5	1	Outside 4D design space	1	–	–	1	–	–
4	Batch at variable conditions			41	−1.0 to 1.0	−1.0 to 1.0	−1.0 to 1.0	1	Conditions at outskirts of DoE 1 design space	–	–	1	–	–	–
5	DoE 2 batches	Doehlert	Two factors: *T*(5)/*F*(3)	35–40	−1.5 to 0.5	1.0	0.2–1.9	8	Includes two center point batches	–	–	–	6	2	–
6	DoE 3 batches	Doehlert	Two factors: *F*(5)/pH(3)	40–44	−1.0	0.4–1.0	−0.5 to 2.5	8	Includes two center point batches	–	–	–	5	3	–
7	Investigative batches			40–44	−1.0	1.0–1.3	1.0–3.0	4	Conditions close to DoE 2 and DoE 3	–	–	–	1	1	2
8	Four reproducibility batches			39–40	−1.0	1.0	2.0	4	Assess batch-to-batch reproducibility	–	–	–	1	2	1
	Total							53		16	8	5	29	12	11

*X*_ind_ biomass concentration at induction of product formation, *T* scaled temperature, *pH* scaled pH, *u*_*C*_ scaled feed rate, *tr* training, *val* validation, *tst* test

#### Design of experiments

At first, a four-factor Doehlert design (DoE 1 in Table 1) with quadratic response surface was applied: 23 experiments (including three center point repeats) with four factors (biomass at induction, induction temperature, pH and feed rate) and two responses: specific and volumetric productivity. Although many different DoE designs are potentially suitable [9, 14, 33, 34], the spherical Doehlert design was selected for its efficiency and the flexibility for subsequent displacement into adjacent experimental regions, in which already carried out experiments can easily be integrated [34]. Furthermore, the Doehlert design offers the possibility to investigate certain factors in more detail than others [35]. In our four-factor design, both biomass at induction and temperature were studied at seven levels, while induction pH and feed rate were studied at five and three levels, respectively [33, 35]. Induction temperature, pH and feed rate were scaled around the center point of DoE 1: *T* = 0; pH = 0 and *u*_*C*_ = 0.

RSM analysis of the 23 DoE batches of DoE1 suggested optimal productivity conditions to be outside of the investigated design space. Therefore, additional batches were performed in experimental regions of lower induction biomass and temperature and higher induction pH and feed rate (DoE 2 and DoE 3 in Table 1). However, experimental results of these additional batches did not obtain the expected higher productivity.

### Hybrid process model

For the described fed-batch *E. coli* process, the material balances provide a sound and general valid modeling framework. The balance equations for biomass and product were derived assuming an ideally mixed reactor, with *X* and *P*/*X* designating biomass and specific productivity, respectively. It is generally assumed that biomass functions as a catalyst in microbial growth and the balance equation for biomass formation is written using a specific rate:

1 $$\frac{{{\text{d}}X}}{{{\text{d}}t}} = \mu \cdot X - D \cdot X$$

where *μ* is the specific biomass growth rate, *D* is a dilution rate equal to $D = (u_{\text{Feed}} + u_{\text{Base}} )/V$ in fed-batch mode. The substrate feeding rate $u_{\text{Feed}}$ and the base addition rate $u_{\text{Base}}$ determine the change in culture volume *V*:

2 $$\frac{{{\text{d}}V}}{{{\text{d}}t}} = u_{\text{Feed}} + u_{\text{Base}}$$

The product is expressed in the cytoplasm, wherefore we introduce an ordinary differential equation for the specific productivity *P*/*X* by:

3 $$\frac{{{\text{d}}\left( {\frac{P}{X}} \right)}}{{{\text{d}}t}} = v_{P/X} \cdot I$$

where $v_{P/X}$ describes the rate of change in the specific productivity and *I* is the induction parameter (0 before induction, 1 after induction). Note that the dilution rate is canceled out in this equation. Equations 1 and 3 can then be combined to obtain the kinetic for volumetric productivity *P* that consists of a growth and non-growth associated product term.

4 $$\frac{{{\text{d}}P}}{{{\text{d}}t}} = \left( {v_{P/X} \cdot I + \frac{P}{X} \cdot \mu } \right) \cdot X - D \cdot P$$

A correlation between biomass growth and base addition was used to exploit the available online base consumption data and to compensate for the relative low number of biomass data points to capture the dynamics of biomass growth, i.e.:

5 $$\mathop \int \limits_{{t_{0} }}^{t} u_{\text{Base}} \cdot {\text{d}}t = a_{\text{Base}} \cdot \left( {X\left( t \right) \cdot V\left( t \right) - X\left( {t_{0} } \right) \cdot V\left( {t_{0} } \right)} \right)$$

where $u_{\text{Base}}$ is the gravimetric base addition rate and $a_{\text{Base}}$ is a function correlating base consumption and biomass growth.

The above-described parametric bioreactor model describes how biomass and specific productivity rates, *μ* and $v_{P/X}$, as well as the base correlation coefficient $a_{\text{Base}}$ are related to biomass and product concentration in time. The rates and correlation coefficient are considered functions of process conditions. They are not directly measurable and therefore they were modeled by means of a nonparametric sub-model as described in the following section.

#### Neural networks

For the approximation of arbitrary nonlinear functions, artificial neural networks are suitable candidates [36, 37] and they have as such been employed in most of the published dynamic serial hybrid modeling structures [23]. Here, the specific rates and base correlation coefficient were approximated by a neural network with three layers. The nodes in the input and output layers of both networks were chosen to have linear transfer functions, whereas the nodes in the hidden layer have tangential hyperbolic transfer functions, $h\left( \cdot \right)$, i.e.:

6 $$\left[ {\mu ,v_{P/X} ,a_{\text{Base}} } \right] = w_{2} \cdot h\left( {w_{1} \cdot x_{1} + b_{1} } \right) + b_{2}$$

where *w*_1_ and *w*_2_ are the weights of the connections between the nodes of the network, *b*_1_ and *b*_2_ are the biases and *x*_1_ is the vector of inputs. For the discrimination of the best network structure and most relevant inputs, as well as for the training/identification of the weights and biases (*w*_1_, *w*_2_ and *b*_1_, *b*_2_, following summarized designated as *w*) each of the data sets was divided into three partitions, a training, validation and test partition, as outlined in the next section.

Different numbers of nodes in the hidden layers were studied for both networks and data sets. The different network structures were compared based on their performance in terms of Bayesian information criteria (BIC), calculated for the training, validation and test data [38]. The best structure was chosen as the one with the greatest BIC value for the validation set, whilst showing consistent performance also for the training set. Different network inputs were studied, the final set of inputs to the artificial neural network comprised *X*, *P*/*X*, *T*, pH and the carbon source feeding rate $u_{C}$, which is the product of $u_{\text{Feed}}$ and the concentration of carbon source in the feed solution.

#### Hybrid modeling datasets

Two datasets were considered for hybrid modeling: one dataset (HM_1_) with experiments of the DoE 1 design space and a larger dataset (HM_2_) comprising all 53 fermentations (Table 1). Each dataset was divided into three partitions: training, validation and test partition. The parameters were identified based on training data, as described in the next section, whereas the validation data were used to determine the point at which the training was stopped, i.e. cross-validation. The test set was used to assess the generalization properties of the models. In HM_1_, DoE 1 batches were split between a training (2/3 of the batches) and a validation partition (1/3 of the batches) in a random fashion. One additional batch was considered in the training partition of HM_1_: a dataset that technically fell just outside the 4D design space, but with each of the individual factors falling within their respective range for DoE 1.

Five batches that allow assessment of model performance with respect to process variations were considered as test batches for HM_1_: four were rejected from the original DoE due to a process control failure that caused feed rate to fluctuate during the induction period, and a fifth batch was performed to challenge the dynamic hybrid model. In this last experiment, temperature, pH and feed rates were varied over the course of the fermentation. The results of these five batches could not be used by the static end-point response surface model, but they could be integrated into the dynamic hybrid semi-parametric model framework, where they proved valuable for assessing the generalization properties of the model. An additional exploratory test set was used with HM_1_, which comprised all fermentations not used during training or validation, to compare HM_1_ performance to HM_2_.

In the case of HM_2_, experiments for which acetate measurements were available were split between the training (2/3 of the data) and validation partition (1/3 of the data) and the remaining data of experiments for which acetate measurements were not available were joined in the test partition. The training set covers different levels of excitation in all process parameters and the validation set contains, repetitions, interpolation and extrapolation cases (Table 1).

#### Parameter identification/weights training

Parameter identification (typically referred to as training in neural network literature) was performed by minimization of the weighted residual of the model estimates, *c*, and the experimental data points *c*_exp_:

7 $$\min_{w} \left\{ {\sum {\frac{{(c_{exp} - c(t,w))^{2} }}{{\sigma_{\text{c}} }}} } \right\},$$

where *σ*_c_ is the variance calculated from the experimental values. The gradient-based minimization algorithm, *lsqnonlin* (Matlab Toolbox) was used. The analytical gradients were obtained using the sensitivity method [20, 22]. The sensitivity equations were integrated along with Eqs. (1–6) using an Euler forward integration method with a fixed step size of 0.25 h. Since a gradient-based identification method was used, the parameter identification was initiated from random weight values at least ten times to find parameter values that approximate the data well. Table 2 summarizes the total number of off-line and on-line data points considered during parameter identification.

**Table 2:** Number of data points of state variables in training, validation and test partitions of HM_1_ and HM_2_

State variable	Property	Hybrid model dataset 1			Hybrid model dataset 2		
		tr	val	tst	tr	val	tst
Off-line data							
*X*	# of points	50	21	17	102	43	43
	Avg # of points/batch	3.1	2.6	3.4	3.5	3.6	3.9
*P/X* and *P*	# of points	40	17	13	95	42	33
	Avg # of points/batch	2.5	2.1	2.6	3.3	3.5	3.0
On-line data							
*u*_*C*_	# of points	884	411	267	1685	661	653
	Avg # of points/batch	55	51	53	58	55	59

## Results and discussion

### Performance of HM_1_ and HM_2_

**Fig. 1:**
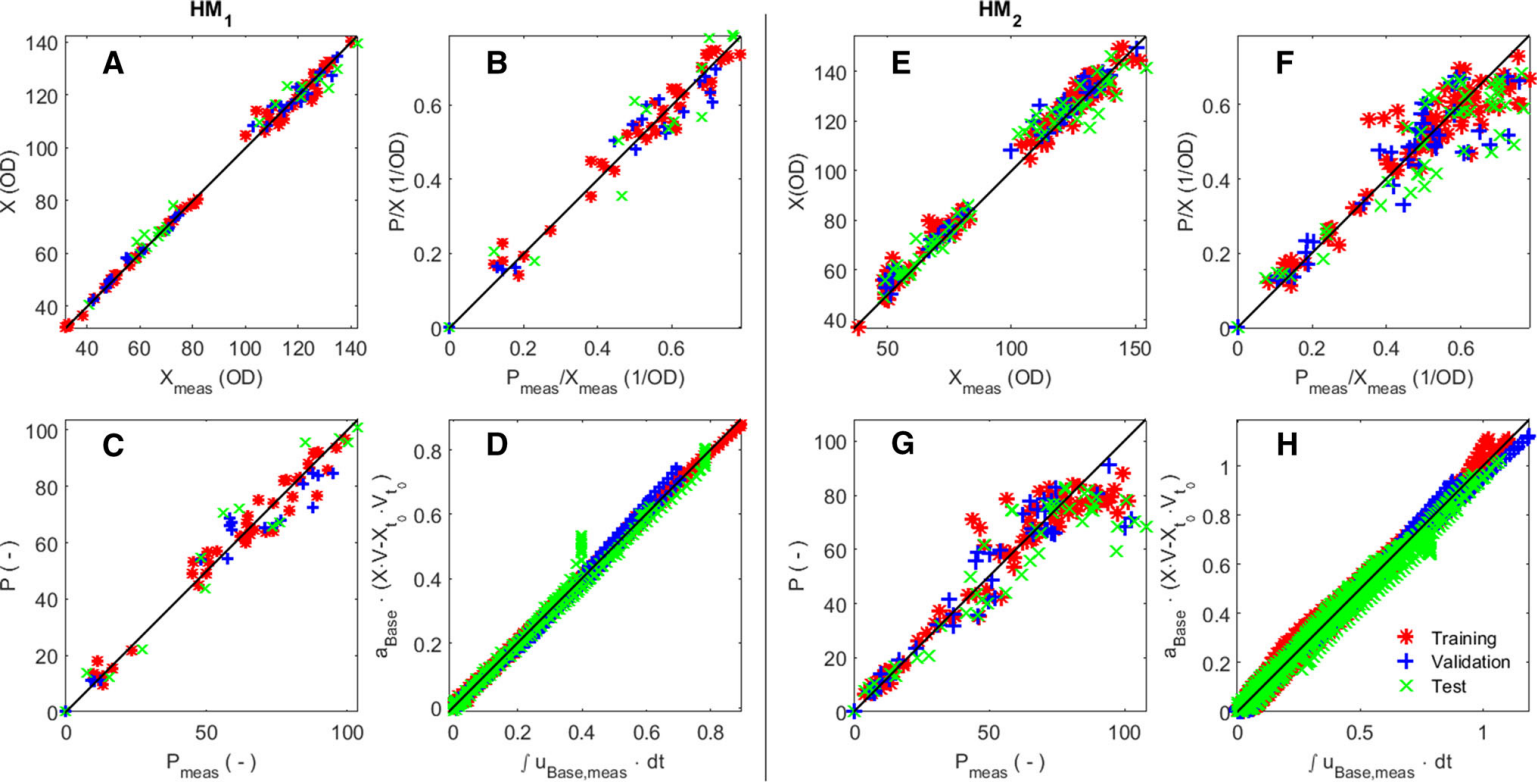
*Left side* **a**–**d** HM_1_ regression plots for biomass, specific and volumetric productivity and for accumulated base addition. *Right side* **e**–**h** HM_2_ regression plots for biomass concentration, specific and volumetric productivity and for the accumulated base addition. Specific and volumetric productivity as well as accumulated base addition were scaled for confidentiality reasons. The training partition is displayed as *red stars*, the validation partition as *blue crosses* and the test partition points are represented by a *green x*. Mathematical symbols as in the *text*

The regression plots of HM_1_ (Fig. 1a–d, left side) show a significant agreement between measured and estimated biomass and product values for the training, validation as well as for the test partition. The significance of agreement for specific and volumetric productivity appears slightly lower than for biomass and is likely caused by (1) a difference in analytical error (biomass measurement requires a single-step dilution vs. multi-step sample treatment for measurement of productivity) and (2) the available high resolution base consumption data correlated directly with biomass formation. The gap in the biomass data between OD 80–100 reflects an absence of overnight sampling.

A significant agreement between measured and estimated points can be observed for the correlation of biomass increase with accumulated base addition for training and validation partitions of HM_1_ (Fig. 1d), which affirms the model performance for biomass. Due to the strong correlation between biomass and frequently logged base consumption, the process dynamics could be captured despite the limited off-line samples taken during each batch. However, one of the batches in the test partition was subject to uncontrolled feed fluctuations outside of the experimental region that caused a deviation in base consumption with respect to biomass growth (see section “Analysis of step-changes on model performance”).

While HM_2_ biomass measurements and estimates are in good agreement (Fig. 1e–h, right side), a deterioration of productivity regression is clearly visible for higher productivity values (*P/X* > 0.375 OD^−1^). To understand the reason for the greater product concentration variance, the (statistical) residual between the measured and predicted product concentration was analyzed, which is described in the next section.

### Product residual analysis

**Fig. 2:**
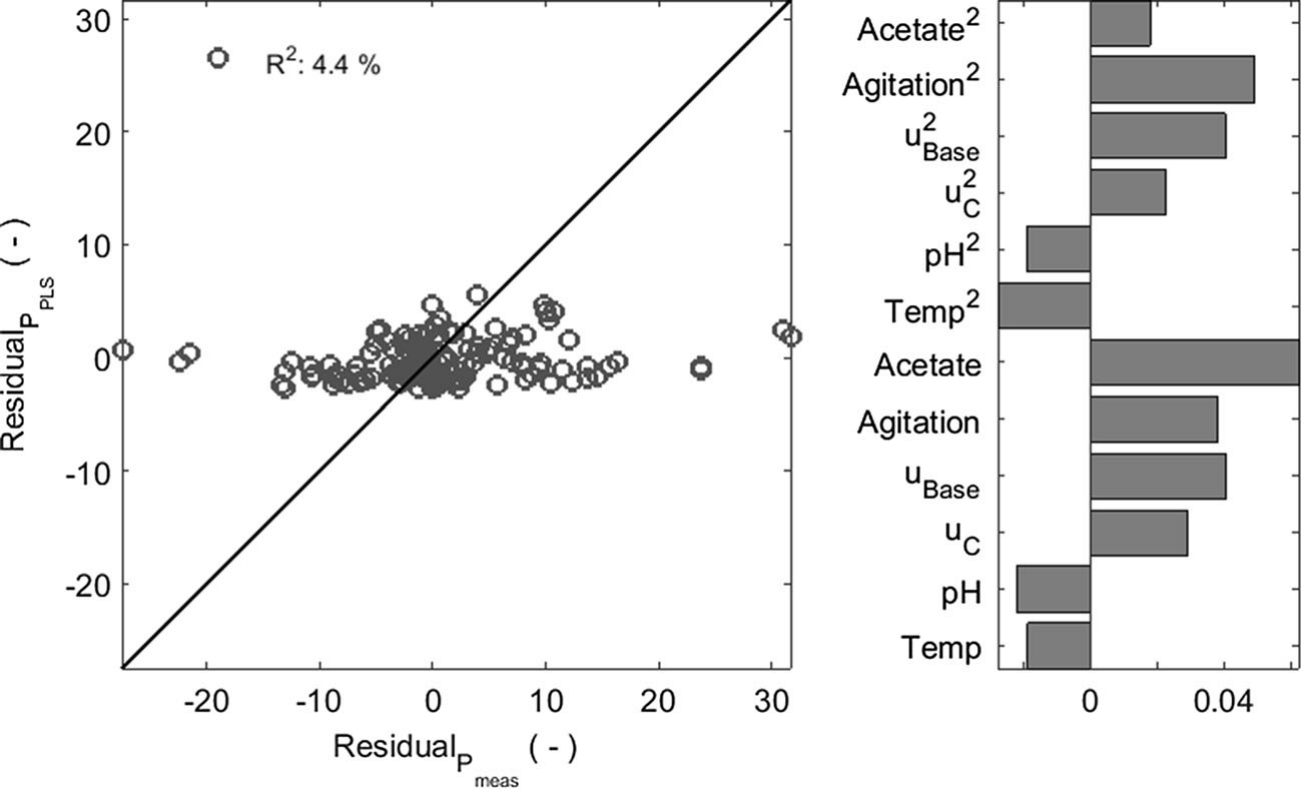
*Left side* Regression plot for residual of the product concentration for HM_2_. *Right side* Regression coefficients that correlate the mean centered and standard deviation scaled process variables to the residual

The residuals of the product concentration (difference between measured and predicted concentration) were analyzed using a partial least square (PLS) method, assessing whether another functional dependency between product concentration and process parameters exists. Besides the ANN inputs *T*, pH and *u*_*c*_, the following variables were also used as input to the PLS: the base addition rate $u_{\text{Base}}$, the agitation rate, the acetate concentration as well as the square values of these variables. The residuals of product concentrations obtained for training and validation partitions of the HM_2_ data set were joined in one data set and randomly partitioned 20 times in sets with 2/3 and 1/3 of the data, each time determining the best number of latent variables using cross-validation. Since acetate concentrations were only measured for experiments included in training and validation partitions of HM_2_, data from the test partition were not included in the analysis. Over 20 repetitions, the number of latent variables that most frequently delivered the best performance was four and subsequently a final PLS model with four latent variables was implemented. The estimation of the PLS for the residuals over the calculated residuals is shown in Fig. 2 together with the impact of the mean centered and standard deviation scaled input variables. It can be seen that the PLS estimates correspond little to the HM_2_ residuals, since only 4.4 % of the variance in product concentration residual can be correlated with tested variables. Thus, (1) most of the functional dependence of the residuals on the included variables is captured by the hybrid model and (2) the following analysis of the impact of the input variables on the residuals must be prudent and supported by additional observations. It can be seen in Fig. 2 that acetate concentration, agitation rate and base addition rate have the greatest impact. These variables were intentionally not included in the nonparametric part (ANN) of the hybrid model. Agitation and base addition rates cannot be independently set as they are implicated in the control of dissolved oxygen concentration and pH, respectively, and acetate is a byproduct of *E. coli* metabolism. Therefore, they are not suitable hybrid model input parameters for process optimization purposes.

Furthermore, measured acetate concentrations are considered to have a relatively high standard variation and were not available for all experiments. High acetate concentrations have been described to affect product formation [32, 39] and several of our cultivations conducted at higher feeding rates were subject to an accumulation of acetate, which might suggest an overflow-like metabolism, similar to the one described by [40]. Additionally, a sudden drop in the stirring rate has been observed during the induction phase of several cultivations (data not shown). The oxygen transfer coefficient (*k*_*L*_*a*) depends on the stirring rate [41, 42] among other factors, and for constant dissolved oxygen concentrations, pressure and temperature the *k*_*L*_*a* is approximately equal to the oxygen uptake rate [43, 44]. This sudden drop in oxygen uptake is likely related to a shift in the physiological state of the cell towards lower oxygen consumption.

### Process model discussion

#### Model validity

**Fig. 3:**
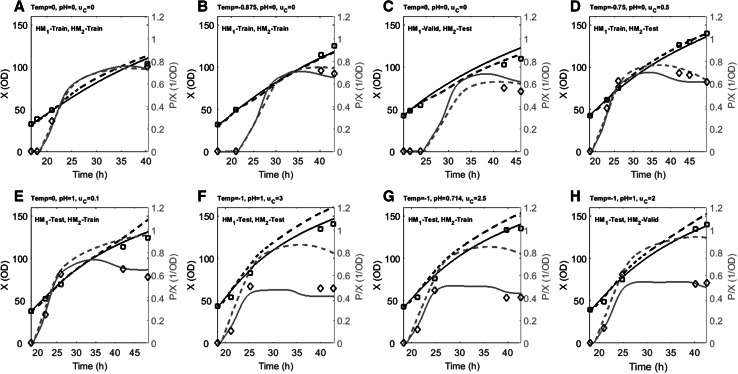
Dynamic profiles for HM_1_ (*dashed lines*) and HM_2_ (*continuous lines*) model estimates for biomass and scaled specific productivity and their respective experimental data points (*squares* biomass, *diamonds* *P/X*). Selected batches at different process conditions that are indicated *above the graphs*. Biomass at induction can be derived from the graph. *Top row* conditions within DoE 1 design space. *Bottom row* conditions outside DoE 1 design space. Note that due to variation in induction periods and sampling frequencies, different time spans are covered for different batches

Dynamic hybrid model profiles of biomass concentration and specific productivity are shown in Fig. 3 for selected process conditions. Batches in Fig. 3a–d are DoE 1 experiments and have close agreement between measured and estimated *X* and *P*/*X* for both HM_1_ and HM_2_. When moving outside this initial design space to lower temperatures, higher pH and higher feed rate, HM_1_ predicts higher specific productivity (Fig. 3e–h), a similar result extrapolated from the RSM analysis on DoE 1 (see “Design of experiments” section). However, batches performed in this extended region did not establish the predicted productivity, hence the creation of HM_2_ that performs better in this region. Due to the respectable modeling performance, the models can be used to analyze the impact of the control degrees of freedom ($X_{\text{ind}} ,T,{\text{pH}}\,{\text{and}}\,u_{c}$) on the process performance.

#### Analysis of process conditions

**Fig. 4:**
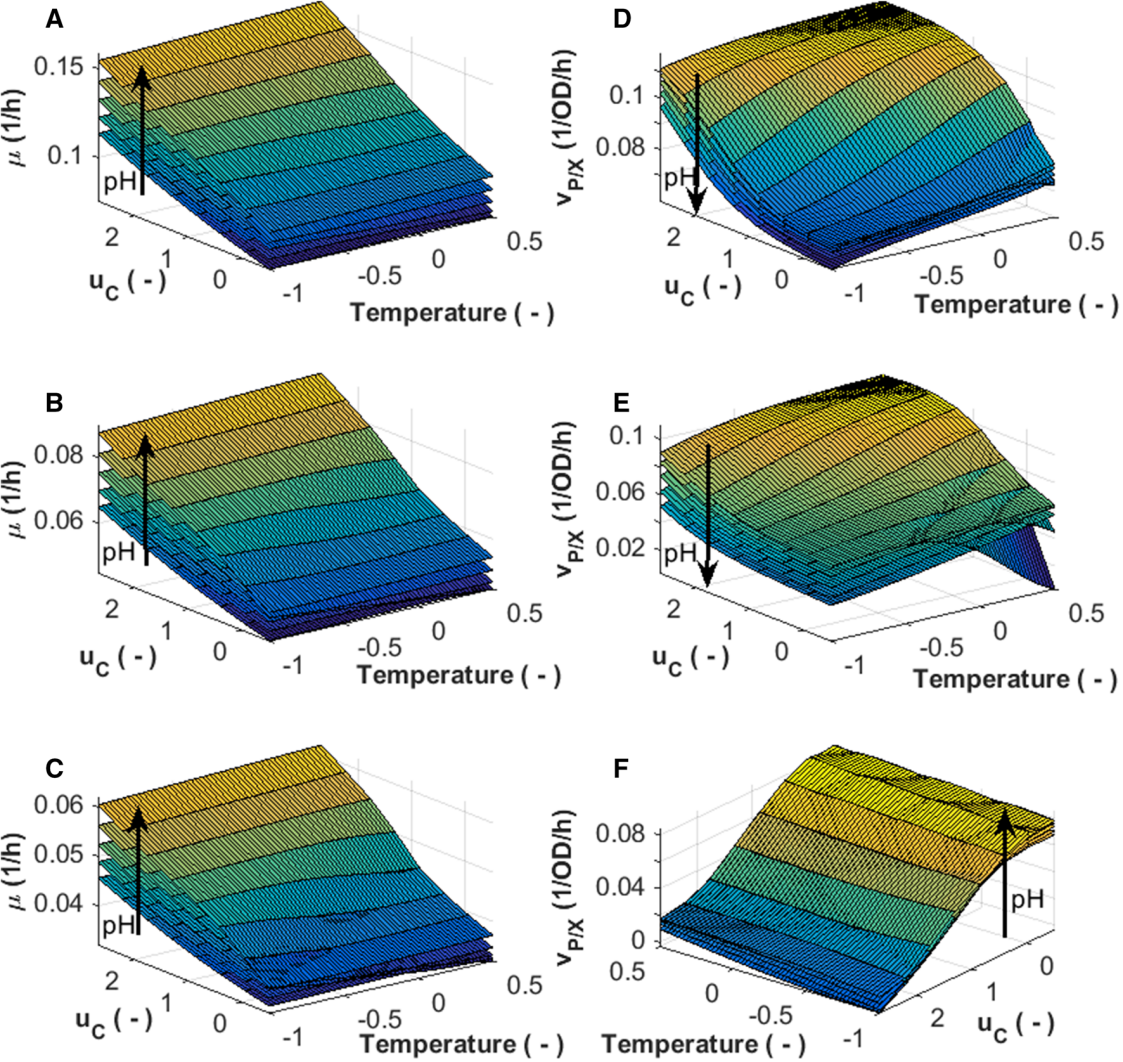
HM_2_ model estimates for specific biomass growth rate (*left side*) and specific productivity rate (*right side*) as function of temperature, pH (−1 to 1 with step size 0.5, the *arrow* indicates increasing pH) and feeding rate at different stages of the process. A&D, early induction: OD 50, *P/X* = 0.125 OD^−1^; B&E, mid induction: OD 80, *P/X* = 0.25 OD^−1^; and C&F, late induction: OD 100, *P/X* = 0.5 OD^−1^. Note that for visibility reasons the *x*- and *y*-axis in *F* are switched and the direction in which feeding rate increases is inverted

Rate dependencies were analyzed to obtain a more detailed view on how process dynamics change with variations in the control degrees of freedom. The impact of changes in temperature, pH and feeding rate on specific biomass growth rate and on specific productivity rate for different biomass concentration and *P/X* levels is shown in Fig. 4. During fed-batch mode, the carbon source is present in concentrations that are growth rate limiting. This is well illustrated by the increasing specific biomass growth rate with increasing feed rate, and the decrease in growth rate with increasing biomass (Fig. 4a–c), which is a common characteristic of fed-batch processes [45]. Temperature does not affect growth significantly which is also plausible under substrate limiting conditions.

Early induction phase conditions are determining product formation; i.e. the specific productivity rate increases with decreasing pH, increasing feed rate and increasing temperature [*P/X* = 0.125 OD^−1^; *X* = 50 (OD) and *P/X* = 0.25 OD^−1^; *X* = 80 (OD), Fig. 4d, e, respectively]. However, with the increase of cytoplasmic expression, the mechanism for product formation seems to be altered [*P/X* = 0.5 OD^−1^; *X* = 100 (OD), Fig. 4f]. According to Glick et al. [46], the metabolism of the host cell can change significantly during heterologous gene expression, often a result of many physiological changes. In our case, specific product formation seems to be negatively affected by the heterologous protein in the cells: i.e. specific productivity rate decreases quickly when specific productivity approaches a concentration of 0.5 OD^−1^ (Fig. 3a–h). Acetate metabolism plays an important role in *E. coli* physiology and high concentrations of this by-product are known to affect recombinant protein production [32, 39, 47]. This may suggest that accumulation of acetate is influencing production kinetics, which would be in agreement with the findings from the residual analysis.

An interesting observation from a process control perspective is that variations in the feeding rate might be compensated by changing parameters such as temperature or pH to yield similar specific productivity kinetics. Thus, multivariate control could help to ensure more consistent process performance.

#### Analysis of step-changes on model performance

The presented modeling approach allows assessment of the impact of temporal process deviations in process variables, in this case temperature, pH, and the feeding rate. Four batches were subject to a process control failure that caused feed rates to fluctuate (Dataset 2 in Table 1). Despite feed rate fluctuations that exceeded the DoE 1 region, biomass and productivity were still estimated well by HM_1_ (illustrated by test partition results in Fig. 1: HM_1_: A–C). For one of these batches, the feed fluctuation led to a pH rise, likely caused by exhaustion of the carbon source causing a similar effect as at the end-of-batch phase (see “Methods”). The increase in pH stopped base consumption, impacting the correlation with biomass growth (Fig. 1: HM_1_: D).

**Fig. 5:**
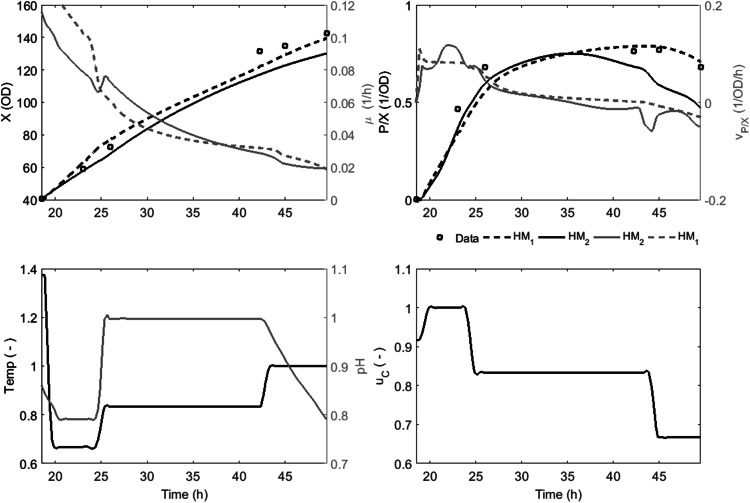
Dynamic batch profiles of a fermentation performed at variable conditions (Dataset #4 in Table 1). *Upper left side* biomass concentration and specific biomass growth rate. *Upper right side* specific productivity and specific productivity rate. *Lower left side* process temperature and pH. *Lower right side* feeding rate

In Fig. 5 the time profiles for biomass and product concentrations and formation rates are displayed for an investigative batch, in which temperature, pH and feeding rate were varied as shown. This batch was included as test batch in both HM_1_ and HM_2_.

Biomass estimates for both HM_1_ and HM_2_ are similar and in agreement with measured biomass values. HM_1_ shows good agreement for the specific productivity: a quick increase in specific productivity followed by a decrease in the specific productivity rate when *P/X* approaches 0.75 OD^−1^. The HM_2_ estimate follows the same trend up to 35 h, after which the estimate moves away from the experimental results, consistent with the observation of higher productivity variability described earlier for HM_2_ (Fig. 1).

The two models suggest that biomass growth rate and specific productivity rate are affected by changes in the process conditions to varying degrees, but the trends of the rates remain the same for both models: i.e. the specific biomass growth rate follows a declining profile that is characteristic for fed-batch cultures and the specific productivity rate is relatively constant at a level of 0.1 OD^−1^ h^−1^ up to a specific productivity of 0.5 OD^−1^ after which it decreases to a second plateau around 0 OD^−1^ h^−1^. Both models show that specific productivity rate turns negative at the end of batch, which could be explained if biomass formation is greater than product formation. The results demonstrate that although the hybrid models were developed for and trained with constant induction set points, they are able to account for changes in conditions.

#### Analysis of the results in relation to PAT and QbD

Understanding the relation between the control parameters and process performance is essential for designing quality into the process. Currently the most wide spread approach in bioprocess development comprises the application of design of experiment methods in combination with response surface type models [15, 17, 18]. The model obtained from this approach, which describes the relation between the control degrees of freedom and process performance, is typically static, assuming that the process parameters are constant throughout the process. Due to the inherent dynamic structure of the described hybrid model, it becomes possible to evaluate the impact of temporal deviations in the control degrees of freedom on the process performance, without performing additional experiments. The increased insight can result in a better understanding of the source and impact of variations, aligning with the call for increased process understanding made in the PAT and QbD approaches. Better understanding of process sensitivities might result in the development of an improved control strategy and/or a strategy for scale-up that accounts for the limitations.

Process time, which becomes available as a control variable with the applied modeling approach, can be taken into consideration when integrating the upstream and downstream processes. This has the potential to mitigate the impact of variations on the overall process performance.

## Conclusions

A hybrid model was developed to describe dynamic biomass growth and recombinant product formation in *E. coli* high cell density fermentation. The model takes into account available theoretical knowledge and experimental data. Two hybrid modeling datasets were generated from 53 *E. coli* high cell density fermentations: a subset of experiments which formed part of a four-factor DoE design (HM_1_) and a set comprising all cultivations (HM_2_). Both models could accurately predict base addition, biomass and product concentrations; however, HM_2_ was less accurate in predicting high product concentrations (*P/X* > 0.375 OD^−1^). An analysis of residuals between measured and predicted productivity did not reveal significant additional functional dependencies, though physiological changes associated with acetate accumulation could have an effect. The correlation between base addition and biomass concentration was beneficial for capturing dynamics of biomass growth, since base consumption data was recorded on-line at high frequency. If off-gas analysis data was available it could similarly be used to correlate with biomass growth and potentially product formation kinetics [27], though as oxygen uptake rate control is less directly achievable, this is less useful for process optimization.

The applied modeling approach enables analysis of impact of control parameters, namely temperature, pH, biomass concentration at induction and feeding rate, on (1) biomass growth and specific productivity rates; and (2) dynamic process profiles. We found that the different process conditions have a significant impact on biomass growth and specific productivity. A change in dependencies of specific productivity rate on process parameters were observed over time, likely a result of physiological changes [46], which could include inhibition at higher specific productivity levels (>0.5 OD^−1^) and acetate accumulation.

With respect to upstream process development and optimization, application of a hybrid modeling approach provides a valuable alternative to conventional statistical analysis. Incorporation of material balances into the hybrid modeling framework provides access to dynamic profiles, which offers the developer the opportunity to take rational decisions with regard to process timings. Functional dependencies of dynamic rates can then be modeled by data-driven methods using data that is typically ignored when applying DoEs with statistical analysis (base accumulation data in our case). The process could be understood in more detail, without the execution of additional experiments, process understanding being at the heart of the PAT and QbD strategy. The possibility to model process performance between offline measurements may be valuable for the improved integration of upstream and downstream processes.

Another benefit of the dynamic approach is that the impact of temporal variations in induction conditions on specific productivity kinetics can be studied and understood, which is a foundational element of PAT. It was, for instance, observed that changes in feeding rate could be compensated by manipulating temperature and/or pH, which in principle enables multivariate process control, and as such fosters progression towards the PAT objective of closed-loop product quality control.
